# Electronic structure and electron-phonon coupling in TiH_2_

**DOI:** 10.1038/srep28102

**Published:** 2016-06-15

**Authors:** K. V. Shanavas, L. Lindsay, D. S. Parker

**Affiliations:** 1 Materials Science & Technology Division, Oak Ridge National Laboratory, Oak Ridge, Tennessee 37831-6056, USA

## Abstract

Calculations using first principles methods and strong coupling theory are carried out to understand the electronic structure and superconductivity in cubic and tetragonal TiH_2_. A large electronic density of states at the Fermi level in the cubic phase arises from Ti-*t*_2g_ states and leads to a structural instability towards tetragonal distortion at low temperatures. However, constraining the in-plane lattice constants diminishes the energy gain associated with the tetragonal distortion, allowing the cubic phase to be stable at low temperatures. Calculated phonon dispersions show decoupled acoustic and optic modes arising from Ti and H vibrations, respectively, and frequencies of optic modes to be rather high. The cubic phase has a large electron-phonon coupling parameter λ and critical temperature of several K. Contribution of the hydrogen sublattice to λ is found to be small in this material, which we understand from strong coupling theory to be due to the small H-*s* DOS at the Fermi level and high energy of hydrogen modes at the tetrahedral sites.

Materials containing light elements such as hydrogen are promising candidates for high temperature superconductors^1^, particularly under pressure when the materials become metallic^2^. Recent discovery of superconductivity above 200 K in H^2^S under pressure^3^ has confirmed these predictions^4^,^5^ and reinvigorated the excitement in the potential of these systems. Metal-hydrogen systems are a class of hydrogen rich materials characterized by large electronic densities at the Fermi level at ambient pressure^6^. Although critical temperatures (*T*_*c*_) are much smaller than hydrogen rich materials under pressure, metal-hydrides such as Th_4_H_15_ (*T*_*c*_ ∼ 8 K) and PdH_x_ (*T*_*c*_ ∼ 11 K) are superconductors at ambient pressure^7^,^8^, and high pressure phases of TiD_0.74_ have been reported to be superconducting near 4 K^9^.

In conventional superconductors, the relevant parameter is the electron-phonon coupling, defined within strong coupling theory^10^ as:


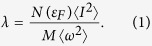


The numerator contains the electronic terms, where *N*(*ε*_*F*_) is electron density at the Fermi level and 〈*I*^2^〉 is the electron-ion matrix element averaged over the Fermi sea. The denominator arises from lattice phonons, *M* is ion mass and 〈*ω*^2^〉 is the average phonon frequency squared^10^. Inspection of Eq. (1) reveals that large electron phonon coupling (and consequently high critical temperatures *T*_c_) is realized in a system with a large density of electronic states at the Fermi level, relatively low phonon energies and/or low ionic mass^6^.

Dihydrides of the form *M*H^2^ (*M* = Ti, Zr, Nb etc.) with face centered cubic structure exhibit a sharp peak in density of states at the Fermi level and have soft phonon modes from the heavy metal atoms, thus making them good candidates for superconductivity^11^,^12^. Unfortunately, the large *N*(*ε*_*F*_) coupled with weak lattice stiffness leads to a martensitic phase transition to a body-centered tetragonal structure below 310 K^13^,^14^. This transition diminishes the density of electrons at the Fermi level. Consequently, AC susceptibility measurements of slightly hydrogen rich TiH_2.07_ found a superconducting transition to be very small, below 15 mK^15^. Theoretical calculations on related compounds ZrH_2_ and NbH_2_ suggested the *T*_*c*_ in these systems to be lower than that of pure metals^16^.

In this manuscript, we present results of first principles calculations carried out to understand the electronic structure and electron-phonon interaction in cubic and tetragonal phases of TiH_2_. We find that constraining the in-plane lattice constants close to 3.13 Å can preserve the cubic phase at low temperatures which we find to have large electron phonon coupling and *T*_*c*_ of several K. Surprisingly, the contribution of H vibrations to *λ* is found to be small in this material and possible reasons are discussed.

## Results

In the cubic phase (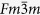) of TiH_2_, the Ti ions occupy a face centered lattice (fcc) with *a* = 4.42 Å in which the H ions occupy tetrahedral sites^17^. The Ti ions are surrounded by eight H ions forming a cube with Ti-H distance of 1.92 Å. Below 310 K, a tetragonal phase is stable in which the unit cell is compressed along *c* and expanded along other directions. In the smallest cell with full symmetry, the Ti ions form a body centered tetragonal (bct) lattice with space group *I*4/*mmm* with *a* = *b* = 3.2 Å and *c* = 4.28 Å (note that, in this cell 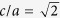 corresponds to the cubic lattice)^18^. Other than a small increase in Ti-H bondlengths to 1.93 Å as a consequence of a small squeeze of TiH_8_ cube along *c*, the bonds remain unchanged.

### Electronic structure

**Figure 1:**
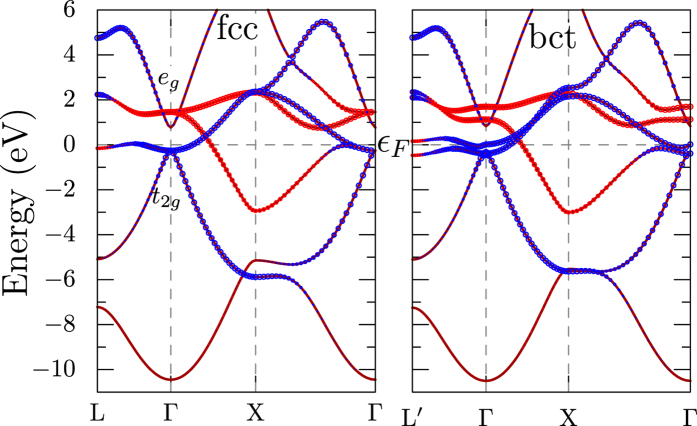
Electronic band structure for the cubic fcc (left) and tetragonal bct (right) phases of TiH_2_. Red and blue colored bands correspond to Ti-*e*_*g*_ and Ti-*t*_*2g*_ characters, respectively.

The band structures for fcc and bct phases are shown in Fig. 1. The bands around −8 eV and +8 eV (not shown) have strong Ti-*s* character which leads to approximately half filled *s* band. In the fcc phase, under the influence of the cubic crystal field, the *e*_*g*_ and *t*_*2g*_ states are split by 1.8 eV at the Γ point with triply degenerate *t*_*2g*_ states occupied just below the Fermi level and the doubly degenerate *e*_g_ states empty. Away from Γ, the *t*_*2g*_ bands couple strongly with H-*s* states and exhibit a large dispersion from −6 to 6 eV. In contrast, the *e*_*g*_ bands are narrower with a bandwidth of 6 eV. The band starting at −5 eV at *L* and increasing along *L* − Γ − *X* has strong H-*s* character and is half filled, indicating 1*s*^1^ configuration for H ions.

The tetragonal distortion breaks the cubic crystal field^19^ and lifts the degeneracy of *t*_*2g*_ into a singlet *d*_*xy*_ band just above ε_*F*_ and a doublet *d*_*xz*_/_yz_ below. The *e*_*g*_ also splits into 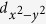 (above) and 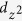 (below). Otherwise, as the figure shows, the dispersion of bands at generic *k*-points are hardly affected by the distortion.

**Figure 2:**
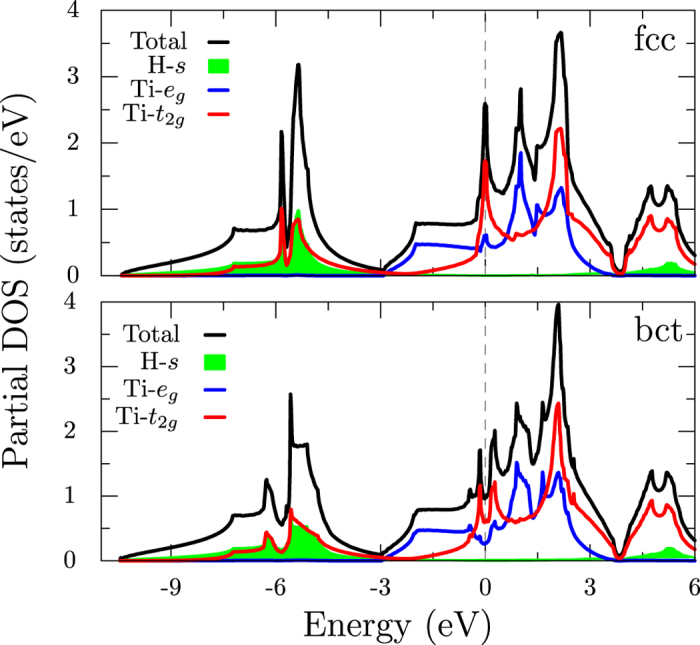
Total and partial density of states for the cubic fcc (top) and tetragonal bct (bottom) phases of TiH_2_. The Fermi level is set to 0 eV. Lines indicate total (black), Ti-*e*_*g*_ (blue) and Ti-*t*_2*g*_ (red) characters while green shaded area is H-*s*.

The total and partial density of states (DOS) plotted in Fig. 2 suggest electronic configuration of 3*d*^3^4*s*^1^ for Ti. The DOS is dominated by a peak around −6 eV mostly containing H-*s* and Ti-*t*_2g_ states and a broad feature around the Fermi level originating from Ti-*d* states. Due to the nearly flat bands in the Γ − *L* direction, there is a large density at the Fermi level *N*(*ε*_*F*_) = 2.59 eV^−1^ per Ti. Most of this density comes from the *t*_2*g*_ states with *N*_*t*2g_(*ε*_*F*_) = 1.92 and *N*_*eg*_(*ε*_*F*_) = 0.67 eV^−1^. Note that the contribution from H-*s* states to the density is quite small at *N*_*s*_(*ε*_*F*_) = 0.001 eV^−1^ per H, which has important implications for superconductivity as will be discussed later. The crystal field in the tetragonal structure breaks the degeneracy of the states and splits the peak in the DOS at *ε*_*F*_ (see Fig. 2). There are two peaks at −0.16 and 0.27 eV with peak heights of 1.7 and 2.0 eV^−1^, respectively. The Fermi level sits in the valley, with *N*(*ε*_*F*_) = 0.97 eV^−1^, which is a more than a 60% drop from that of the cubic phase. Since the electron-phonon coupling strength is proportional to *N*(*ε*_*F*_), we can see why the tetragonal phase is not a favorable candidate for superconductivity.

**Figure 3:**
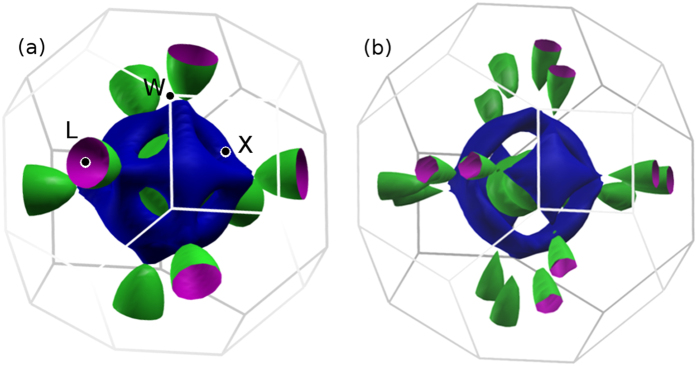
Calculated Fermi surfaces for the (**a**) fcc and (**b**) bct phases corresponding to the two bands that cross the Fermi level.

The Fermi surfaces for the two phases of TiH_2_ are shown in Fig. 3. As can be seen from the band structure plots in Fig. 1, two bands cross the Fermi level, leading to large electron pockets around Γ. In the tetragonal phase, the band splittings lead to additional features in the Fermi surfaces. Figure 3 suggests that Fermi surfaces in TiH_2_ are not amenable to nesting induced instabilities.

The Jahn-Teller (JT) theorem stipulates that a non-linear molecule with degenerate electronic ground states will be unstable against symmetry lowering distortion^20^. For a single electron in doubly degenerate states, JT distortion *Q* lowers the energy of one of the states by −*gQ* and the equilibrium configuration is decided by the competition between this and an elastic term of the form *KQ*^2^/2, where *g* and *K* are proportionality constants. In a solid, the expression also involves a positive band energy term since electron hopping prefers degenerate states^21^. It can be shown that, JT distortion occurs in a material when the coupling parameter *g* is sufficiently strong, viz., *g*^2^/(*WK*) > *μ*_*c*_, where *W* is the bandwidth and *μ*_*c*_ is the critical limit^21^, which is *μ*_*c*_ ∼ 0.5 for a two state, one electron system discussed above. Incidentally, in systems with heavy elements such as HfH_2_, spin-orbit coupling partially lifts the degeneracy of the levels near *ε*_*F*_^14^, but the system remains JT active, since the distortion *Q* can further lower the energy.

**Figure 4:**
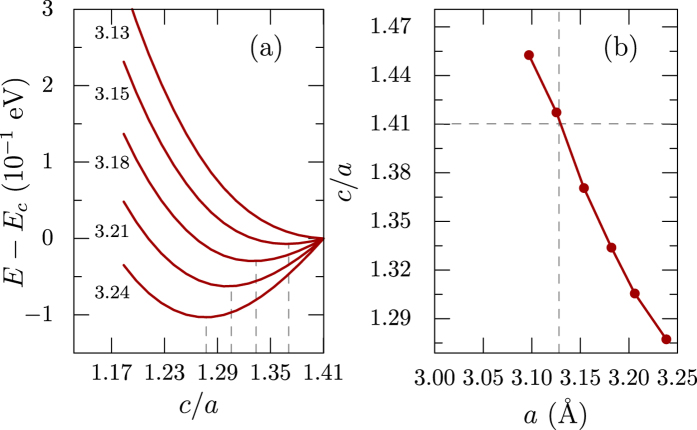
(**a**) Energy variation of the system as function of *c*/*a* ratio for fixed in-plane lattice constant *a* as marked left of the curves. The energy is shifted by its value when 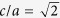 (*E*_*c*_). The minima of the curves are marked by dashed vertical lines. (**b**) Variation of *c*/*a* corresponding to minimum energy as a function of *a*. The ratio 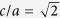 corresponds to the cubic phase.

In the relaxed structure within GGA, the lattice constants of TiH_2_ tetragonal phase are *a* = *b* = 3.21 Å and as can be seen from Fig. 4(a), the energy is minimum for 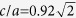. Decreasing *a* shifts the minimum to higher *c*/*a* and for *a* = 3.13 Å the optimal 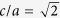, making the cubic phase stable. As shown in Fig. 4(b), the ratio *c*/*a* decreases almost linearly as the in-plane lattice constants are increased. Thus, constraining the in-plane lattice constants close to *a* = *b* = 3.13 Å can shift the stable phase of TiH_2_ close to cubic, which may be achievable in experiments by growing the material on a suitable substrate with appropriate lattice constants.

### Electron-phonon coupling

**Figure 5:**
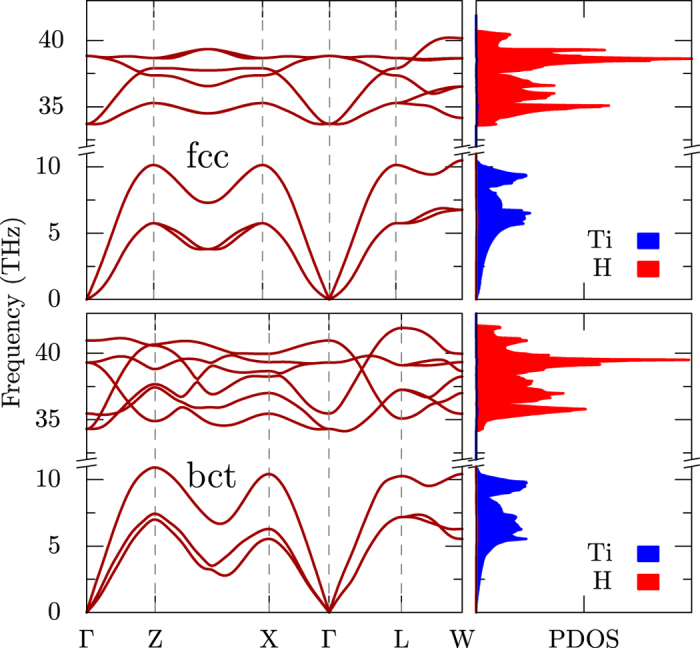
The phonon dispersions and density of states for cubic (left) and tetragonal (right) phases of TiH_2_. Electronic smearing of *σ* = 0.2 eV is used to remove the negative frequencies in the cubic phase. Partial DOS for Ti (blue) and H (red) are plotted in the right panel.

The phonon dispersion curves and corresponding densities of states for the cubic and tetragonal structures in the experimental structures calculated using density functional perturbation theory are plotted in Fig. 5. As expected, the acoustic dispersions have predominantly Ti character while the optic modes that lie between 33–40 THz originates from hydrogen vibrations. We find soft modes in the phonon dispersion curves of the cubic phase around the Γ point, which can be suppressed by choosing a larger temperature smearing, *σ*, of electronic states near the Fermi level^12^. Figure 5 is calculated with *σ* = 0.2 eV, which barely removes the negative frequencies in the fcc phase (*σ* = 0.14 eV is used for the bct phase). Similar to the electronic band structure, the reduced symmetry in the tetragonal phase lifts the degeneracies of dispersions at high symmetry points, but leaves very similar phonon density of states in both structures.

**Figure 6:**
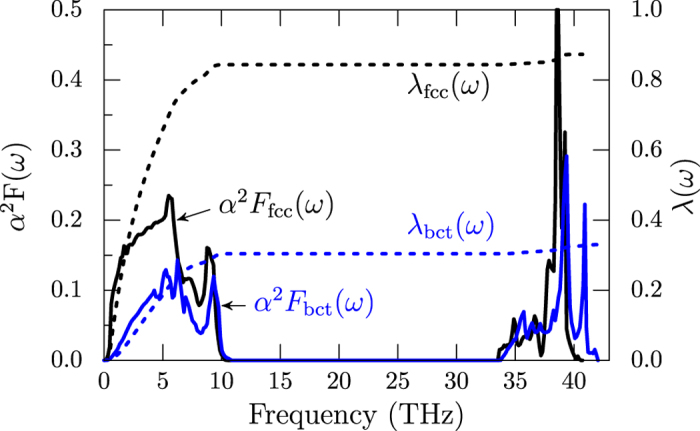
Calculated Eliashberg spectral function *α*^2^*F*(*ω*) (solid lines) and electron-phonon coupling parameter *λ*(*ω*) (dashed lines) for fcc (black) and bct (blue) phases.

To get the strength of coupling between electrons and phonons, we calculated the Eliashberg spectral function *α*^2^*F*(*ω*) using QE package for fcc and bct phases, that are shown in Fig. 6. We find that in both cases the function has two main peaks; one below 10 THz and another between 33–42 THz. The low energy peak arises from the acoustic modes, while the high energy peak arises from optic modes that are nearly decoupled in these systems. As a consequence of the reduction of *N*(*ε*_*F*_) in the case of the tetragonal phase *α*^2^*F*(*ω*) is smaller. The electron-phonon coupling parameter, 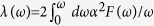, is also plotted as dotted lines in Fig. 6, which show that Ti modes contribute predominantly towards *λ*. In the cubic phase, we get *λ*_fcc_ = 0.87, which drops to *λ*_bct_ = 0.33 in the bct phase. More accurate estimate of *λ* values calculated by summing over the reciprocal space *λ*(*q*) yield, *λ*_fcc_ = 0.84 and *λ*_bct_ = 0.22. Using these values in the simplified Allen-Dynes formula^22^ to calculate the *T*_*c*_ with the typical value of μ* = 0.1 for the Coulomb coefficient^23^, we get *T*_*c*_ = 6.7 K in the cubic phase, which drops to *T*_*c*_ = 2 mK in the tetragonal phase. In comparison, the experiments on TiH_2.07_ report superconducting behavior below 15 mK^15^, which is reasonably close to our calculated value for bct phase. We note that the *λ*_fcc_ is comparable to that of MgB_2_, which is superconducting at 40 K. The lower *T*_*c*_ here is because of the smaller log mean frequency *ω*_log_ in TiH_2_, which is 127.1 K, compared to *ω*_log_ = 650 K in MgB_2_^24^.

The contribution of hydrogen modes to electron-phonon coupling and consequently to *T*_*c*_ is much smaller than other hydrides such as PdH and H_3_S, where hydrogen plays a dominant role^16^,^25^. To understand this, we calculate *λ* using McMillan’s strong coupling theory^10^ given in Eq. (1). Since the acoustic and optic modes have negligible hydrogen and metal character respectively, we can write *λ* = *λ*_T*i*_ + 2*λ*_H_. We calculate the numerator in Eq. (1), *η* = *N*(*ε*_*F*_)〈*I*^2^〉, with the help of Gaspari-Gyorffy theory^26^,


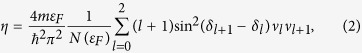


where, 
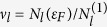
 is the ratio of the *l*^th^ partial DOS at Fermi energy to single-site DOS at Fermi energy and *δ*_*l*_ are the scattering phase shifts for the angular momentum *l*. Taking the calculated phase shifts and partial densities for cubic TiH_2_ from ref. 27, we get: *η *_T*i*_ = 3.9 eV/Å^2^ and *η*_H_ = 0.07 eV/Å^2^. The *η*_H_ is much smaller than that of Ti in this system compared to PdH where *η*_Pd_/*η*_H_ = 1.38^16^. It is a consequence of much smaller H-*s* density at the Fermi level in TiH_2_, which is only 0.001 eV^−1^ (Fig. 2), in contrast to PdH, where it is 0.019 eV^−1^.

The renormalized phonon frequencies 〈*ω*^2^〉 are calculated using the definitions in ref. 10 and our phonon data to get, 〈*ω*^2^〉_Ti_ = 6.72 × 10^26^ (rad/sec)^2^ and 〈*ω*^2^〉_H_ = 5.32 × 10^28^ (rad/sec)^2^. The frequencies of hydrogen modes in TiH_2_ are higher than those in other hydrogen rich systems, which also lead to the smaller contribution of hydrogen modes to *λ* in this system. Putting everything together, we get for the cubic phase of TiH_2_:


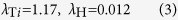


Adding up, we get *λ*_fcc_ = 1.19 from strong coupling theory, which overestimates *λ* from our first principles calculated value of 0.84. However, considering the crudeness of the strong coupling model, the agreement is reasonable. Crucially, it explains the insignificant contribution from hydrogen in this system, due to a small density of H electronic states at the Fermi energy coupled with unusually high energies of the hydrogen sublattice vibrations in TiH_2_. In H_3_S, the *N*(*E*_*F*_) at high pressures, although small, is dominated by H-*s* states and is crucial for the reported high *T*_*c*_. To enhance the *T*_*c*_ in transition metal hydrides such as TiH_2_, will likely require manipulating the electronic structure via pressure, doping or replacing Ti with other transition metal ions to increase the H-*s* density near the Fermi level. We hope that this work inspires further studies to this end.

## Discussion

In summary, our theoretical calculations show that the cubic phase of TiH_2_ has large electron density at the Fermi level arising from nearly flat triply degenerate Ti-*t*_2g_ bands in the Γ − *L* direction. This large density of *N*(*ε*_*F*_) = 2.59 eV^−1^ combined with relatively weak structural elastic energy, leads to a cubic-to-tetragonal distortion at low temperatures. However, constraining the in-plane lattice constants to 3.13 Å makes the cubic geometry energetically favorable, which may be possible to achieve by a suitably lattice matched substrate. The calculated phonon dispersions for the cubic and tetragonal phases show many similarities owing to the smallness of the distortion. We estimate that, *λ*_fcc_ = 0.84, *T*_*c*_ = 6.7 K for the cubic phase and *λ*_bct_ = 0.22, *T*_*c*_ =2 mK for the tetragonal phase. The contribution of hydrogen modes to the electron-phonon coupling is small, which is a consequence of small H-*s* density at the Fermi level and unusually hard hydrogen modes in this system. Alloying with other transition metals and with isotopes of hydrogen can potentially enhance the contribution from the hydrogen sublattice, by shifting the H-*s* levels and lowering optic modes, respectively, and require further investigations.

## Methods

First principles calculations within density functional theory and the generalized gradient approximation (GGA)^28^ are employed. The VASP code^29^,^30^ with projector augmented waves as basis is used for the electronic structure calculations. An energy cutoff of 450 eV and *k* space sampling on a 24 × 24 × 24 grid are found sufficient to get converged results. The phonon dispersions and electron-phonon couplings are obtained using the Quantum Espresso package^31^ with ultrasoft pseudopotentials, energy cutoff of 544 eV (40 Ry) and a *q*-grid of 8 × 8 × 8.

## Additional Information

**How to cite this article**: Shanavas, K. V. *et al*. Electronic structure and electron-phonon coupling in TiH_2_. *Sci. Rep.* **6**, 28102; doi: 10.1038/srep28102 (2016).
